# The Richness and Diversity of Catalases in Bacteria

**DOI:** 10.3389/fmicb.2021.645477

**Published:** 2021-03-19

**Authors:** Fang Yuan, Shouliang Yin, Yang Xu, Lijun Xiang, Haiyan Wang, Zilong Li, Keqiang Fan, Guohui Pan

**Affiliations:** ^1^State Key Laboratory of Microbial Resources, Institute of Microbiology, Chinese Academy of Sciences, Beijing, China; ^2^University of Chinese Academy of Sciences, Beijing, China; ^3^School of Life Sciences, North China University of Science and Technology, Tangshan, China

**Keywords:** catalase, hydrogen peroxide, monofunctional catalase, catalase-peroxidase, manganese-containing catalase, bacteria, *Streptomyces*, secondary metabolites

## Abstract

Catalases play a key role in the defense against oxidative stress in bacteria by catalyzing the decomposition of H_2_O_2_. In addition, catalases are also involved in multiple cellular processes, such as cell development and differentiation, as well as metabolite production. However, little is known about the abundance, diversity, and distribution of catalases in bacteria. In this study, we systematically surveyed and classified the homologs of three catalase families from 2,634 bacterial genomes. It was found that both of the typical catalase and Mn-catalase families could be divided into distinct groups, while the catalase-peroxidase homologs formed a tight family. The typical catalases are rich in all the analyzed bacterial phyla except Chlorobi, in which the catalase-peroxidases are dominant. Catalase-peroxidases are rich in many phyla, but lacking in Deinococcus-Thermus, Spirochetes, and Firmicutes. Mn-catalases are found mainly in Firmicutes and Deinococcus-Thermus, but are rare in many other phyla. Given the fact that catalases were reported to be involved in secondary metabolite biosynthesis in several *Streptomyces* strains, the distribution of catalases in the genus *Streptomyces* was given more attention herein. On average, there are 2.99 typical catalases and 0.99 catalase-peroxidases in each *Streptomyces* genome, while no Mn-catalases were identified. To understand detailed properties of catalases in *Streptomyces*, we characterized all the five typical catalases from *S. rimosus* ATCC 10970, the oxytetracycline-producing strain. The five catalases showed typical catalase activity, but possessed different catalytic properties. Our findings contribute to the more detailed classification of catalases and facilitate further studies about their physiological roles in secondary metabolite biosynthesis and other cellular processes, which might facilitate the yield improvement of valuable secondary metabolites in engineered bacteria.

## Introduction

Reactive oxygen species (ROS) are generated in the O_2_ reduction process, including superoxide anion (O_2_^⋅–^), hydrogen peroxide (H_2_O_2_), and hydroxyl radical (OH^⋅^) (Montibus et al., 2015; Johnson and Hug, 2019; Kim et al., 2019). These ROS can cause the oxidative damage of cellular macromolecules and lead to dysfunction of cells (Montibus et al., 2015; Johnson and Hug, 2019). Due to the pervasive threat of ROS, bacteria have evolved various scavenging and defense enzymes to mitigate this threat (Johnson and Hug, 2019; Kim et al., 2019). Catalases are one of the families of well-known ROS defense enzymes, which catalyze the decomposition of H_2_O_2_ (Nicholls et al., 2000; Chelikani et al., 2004; Zamocky et al., 2008; Nicholls, 2012; Johnson and Hug, 2019). In nature, there are three families of catalases with different protein sequence characteristics and co-factor dependences, including the heme-containing monofunctional typical catalases, heme-containing bifunctional catalase-peroxidases, and manganese-containing catalases (Mn-catalases) (Chelikani et al., 2004; Zamocky et al., 2008; Johnson and Hug, 2019).

The typical catalases catalyze dismutation of H_2_O_2_ through a two-stage mechanism (Reactions 1 and 2) (Chelikani et al., 2004; Zamocky et al., 2008; Sooch et al., 2014). In the first stage, the heme iron in the active center is oxidized by a molecule of H_2_O_2_ to form the intermediate oxoferryl porphyrin π-cation radical (^⋅^^+^Por-Fe(IV)=O, compound I), together with the production of water (Reaction 1). In the second stage, compound I is rapidly reduced back to ferrous porphyrin form by H_2_O_2_, with the formation of molecular oxygen and water (Reaction 2). However, the detailed mechanism of the two-electron reduction of compound I by H_2_O_2_ was still not very clear (Alfonso-Prieto et al., 2012). Previous studies proposed that the reduction process involved the transfer of a hydride ion from H_2_O_2_ to compound I (Fita and Rossmann, 1985; Kato et al., 2004; Alfonso-Prieto et al., 2012), while the metadynamics simulation analysis suggested that the transfer of one hydrogen atom from H_2_O_2_ to compound I occurred first, followed by another reduction reaction (Kato et al., 2004; Alfonso-Prieto et al., 2009, 2012).

$$\mathrm{Por}\text{-}\mathrm{Fe}\left(\mathrm{III}\right)+H_{2}O_{2}\to^{\cdot +}\mathrm{Por}\text{-}\mathrm{Fe}\left(\mathrm{IV}\right)=O+H_{2}O$$

(Reaction 1)

Por⋅+-Fe(IV)=O+H2O2→Por-Fe(III)+O2+H2O

(Reaction 2)

The bifunctional catalase-peroxidases catalyze dismutation of H_2_O_2_, and decomposition of H_2_O_2_ using some unidentified organic electron donors (Chelikani et al., 2004; Zamocky et al., 2008). The catalytic mechanism of catalase-peroxidase is complicated and involves several intermediates (Zamocky et al., 2008; Vlasits et al., 2010). Briefly, the heme iron is first oxidized by H_2_O_2_ to form compound I (Reaction 1). Compound I can be reduced to compound II (Por-Fe(IV)-OH) via transfer of a hydrogen atom from organic electron donors (Reaction 3), followed by another hydrogen atom transfer with the production of water (Reaction 4). In the absence of exogenous electron donor, the porphyrin radical of compound I can be quenched by an electron transferred from protein residues forming compound I^∗^ (^⋅ +^ AA Por-Fe(IV)-OH; Reaction 5) (Zamocky et al., 2008; Vlasits et al., 2010), which is reduced by another molecule of H_2_O_2_ to form compound III^∗^ ([^⋅ +^ AA Por-Fe(II)-O_2_ ↔ ^⋅ +^ AA Por-Fe(III)-O_2_^⋅–^]; Reaction 6) (Vlasits et al., 2010). After the release of molecular oxygen, the enzyme returns to its ferric form (Reaction 7).

Por⋅+-Fe(IV)=O+AH2→Por-Fe(IV)-OH+AH⋅

(Reaction 3)

Por-Fe(IV)-OH+AH2→Por-Fe(III)+AH⋅+H2O

(Reaction 4)

$${\mathrm{AA}}^{\cdot +}\mathrm{Por}\text{-}\mathrm{Fe}\left(\mathrm{IV}\right)=O+H^{+}\to^{\cdot +}\mathrm{AA}\mathrm{Por}\text{-}\mathrm{Fe}\left(\mathrm{IV}\right)-\mathrm{OH}$$

(Reaction 5)

AA⋅+Por-Fe(IV)-OH+H2O2→[⋅+AAPor-Fe(II)-O2

$${↔}^{\cdot +}\mathrm{AA}\mathrm{Por}\text{-}\mathrm{Fe}\left(\mathrm{III}\right)-O_{2}^{\cdot -}]+H{}_{2}O+H{}^{+}(\mathrm{Reaction6})$$

$${[}^{\cdot +}\mathrm{AA}\mathrm{Por}\text{-}\mathrm{Fe}\left(\mathrm{II}\right)-O_{2}{↔}^{\cdot +}\mathrm{AA}\mathrm{Por}\text{-}\mathrm{Fe}\left(\mathrm{III}\right)-O_{2}^{\cdot -}]\to$$

AAPor-Fe(III)+O2(Reaction 7)

The Mn-catalases are found mainly in bacteria and archaea, which catalyze dismutation of H_2_O_2_ but with relatively low specific activities compared to the heme-containing typical catalases (Chelikani et al., 2004; Zamocky et al., 2008; Whittaker, 2012). The Mn-catalase has a unique di-manganese cluster (Mn^2+^-Mn^2+^ or Mn^3+^-Mn^3+^) in the active center, and catalyzes a different two-stage reaction (Chelikani et al., 2004; Zamocky et al., 2008; Whittaker, 2012; Sooch et al., 2014). The Mn^2+^-Mn^2+^ cluster is first oxidized by a molecule of H_2_O_2_ to form the Mn^3+^-Mn^3+^ cluster and two molecules of water (Reaction 8). And then, the Mn^3+^-Mn^3+^ cluster is reduced by another molecule of H_2_O_2_ and releases molecular oxygen (Reaction 9) (Chelikani et al., 2004; Zamocky et al., 2008).

$${\mathrm{Mn}}^{2+}\text{-}{\mathrm{Mn}}^{2+}+2H^{+}+H_{2}O_{2}\to {\mathrm{Mn}}^{3+}\text{-}{\mathrm{Mn}}^{3+}$$

+2H2O(Reaction 8)

$${\mathrm{Mn}}^{3+}\text{-}{\mathrm{Mn}}^{3+}+H_{2}O_{2}\to {\mathrm{Mn}}^{2+}\text{-}{\mathrm{Mn}}^{2+}+2H^{+}$$

+O2(Reaction 9)

As the major H_2_O_2_ scavenger, catalases play an important role in the defense against oxidative stress and the maintenance of intracellular H_2_O_2_ concentrations in bacteria (Montibus et al., 2015; Johnson and Hug, 2019). It is common that multiple catalases are encoded in one bacterium (Mulvey et al., 1990; Engelmann and Hecker, 1996; Cho et al., 2000; Klotz and Loewen, 2003) and they have been shown to behave differently in response to oxidative stress in some studies (Chelikani et al., 2004; Zamocky et al., 2008). For example, in *E. coli*, the catalase-peroxidase KatG was mainly expressed upon induction of oxidative stress, whereas the typical catalase KatE (HPII) was induced in the stationary growth phase (Mulvey et al., 1990; Zamocky et al., 2008). Studies have also showed that manipulating the expression of catalases could affect the development and secondary metabolite production in strains like streptomycetes (Cho et al., 2000; Beites et al., 2011). Disruption of *catB* (encoding a typical catalase) in *S. coelicolor* caused impairment in the formation of aerial mycelium and resistance against osmotic stress, and interestingly led to the reduced production of undecylprodigiosin and hyperproduction of actinorhodin (Cho et al., 2000). On the contrary, disruption of *catA* (encoding a typical catalase) showed no observed effect on the sporulation of *S. coelicolor*, but significantly reduced its tolerance to H_2_O_2_ stress (Cho et al., 2000). These previous studies highlighted the important physiological roles of catalases in bacteria. However, there is a lack of systematic understanding of the richness, diversity, and distribution of catalases in bacteria. Previous studies briefly surveyed the catalases in bacteria, archaea and eukarya, but only a very limited number of catalase homologs (usually 100-200 homologs for each family) were included for the analysis at that time (von Ossowski et al., 1993; Klotz et al., 1997; Klotz and Loewen, 2003; Chelikani et al., 2004; Zamocky et al., 2008; Zámocký et al., 2012).

In this study, we systematically surveyed and analyzed the homologs of three catalase families in bacterial genomes, which enabled us to reveal the richness, diversity, and taxonomic distribution of catalases in bacteria. We further characterized five typical catalases from one representative *Streptomyces* strain, all of which could catalyze the decomposition of H_2_O_2_ but with varied catalytic properties. Our study has provided the basis for further investigation of bacterial catalases to better understand their physiological roles in various cellular processes.

## Materials and Methods

### Bacterial Strains and Growth Conditions

*S. rimosus* ATCC 10970 was obtained from China General Microbiological Culture Collection Center (CGMCC 4.1438). *E. coli* JM109 and BL21(DE3) were used for general cloning and protein expression, respectively. LB medium was used for *E. coli* cultivation. Kanamycin was used at a final concentration of 50 μg/mL. Restriction enzymes and Q5 DNA polymerase were purchased from New England Biolabs (United States). DNA manipulations, competent cell preparation, and transformation were performed as described previously (Sambrook and Russell, 2001). Hydrogen peroxide (30%) solution was purchased from Sigma-Aldrich. The 96-well UV-transparent microplates were purchased from Corning.

### Expression and Purification of the Five Catalases

The plasmids and primers used in this study are listed in Supplementary Tables 1, 2, respectively. The five catalase genes of *S. rimosus* ATCC 10970 were amplified through PCR with their corresponding primer pairs (Supplementary Table 2) using the genomic DNA of *S. rimosus* as a template. The plasmid pET28a was digested by *Nde*I and *Xho*I, and then joined with each of the five catalase gene fragments by Gibson assembly (Gibson et al., 2009) to generate the five overexpression plasmids (Supplementary Table 1). All the plasmids were verified by DNA sequencing and transformed into *E. coli* BL21(DE3) to obtain the five overexpression strains.

The strains were cultivated in LB supplemented with 50 μg/mL of kanamycin at 37°C and 220 rpm until an OD600 of 0.6 was reached, at which time final concentrations of 100 μM isopropyl-β-D-thiogalactoside (IPTG), 0.5 mM 5-aminolevulinic acid, and 0.2 mM FeCl_3_ were added. The cell cultures were further cultivated for 8 h at 28°C and 180 rpm. The five N-terminal His_6_-tagged catalases (Cat1-5) were purified by Nickel-NTA affinity chromatography according to the previously reported protocol (Fan et al., 2012), concentrated by centrifugation, and exchanged to ultrafiltration buffer (50 mM Na_2_HPO_4_-NaH_2_PO_4_, 10% (v:v) glycerol, pH 7.4) using 10-kDa Amicon Ultra tubes (Centriplus, Merck Millipore). The purities of the proteins were evaluated by 10% SDS-PAGE. The protein concentrations were determined by Bradford assay using bovine serum albumin (BSA) as a standard. The proteins were stored at –70°C.

### Heme Occupancy and Thermostability of the Five Catalases by Spectroscopic Analysis

The UV-vis spectra of five catalases were collected on a Beckman Coulter DU800 spectrophotometer using a cell with an optical path of 1 cm. The heme concentrations were calculated using absorption at 406 nm (ε_406_ = 102 mM^–1^ cm^–1^) (Brill and Sandberg, 1968). The heme occupancy of each catalase was the ratio of heme concentration to the corresponding protein concentration (by Bradford assay).

Circular dichroism (CD) spectra of five catalases were monitored in the far-UV range (195–260 nm) in 50 mM Na_2_HPO_4_-NaH_2_PO_4_ (pH 7.4), at 30°C on a Chirascan^TM^ CD Spectrometer (Applied Photophysics, United Kingdom) using a cell with an optical path of 1 mm. The stability of the five catalases were monitored by CD signals at 222 nm (representing the content of α-helices) from 20 to 90°C (raising 1°C at a time, and holding for 5 s before data collection) in 50 mM Na_2_HPO_4_-NaH_2_PO_4_ (pH 7.4).

### Enzymatic Reactions of the Five Catalases

The activities of the five catalases were measured using the spectrophotometric method described previously (Beers and Sizer, 1952). A typical 200 μL reaction mixture consisted of 16 mM H_2_O_2_, purified catalase (1.06-10.32 nM), and 50 mM Na_2_HPO_4_-NaH_2_PO_4_ (pH 7.4). The absorbance at 240 nm (representing the concentration of H_2_O_2_) was monitored continuously in 96-well plates at 30°C using an EnSpire^TM^ multimode Plate Reader (PerkinElmer Inc., United States). The reaction rates were calculated using the absorbance changes at 240 nm within the first 1 min, and averaged for three independent reaction samples. One unit of enzyme activity is defined as the amount of activity required for the conversion of 1 μmol of H_2_O_2_ into water and oxygen per minute at 30°C (Zeng et al., 2011).

The catalase activities under different pH conditions were measured at 30°C. A series of buffers were used, including 50 mM NaAc-HAc (pH 4.0 – 6.0), 50 mM NaH_2_PO_4_-Na_2_HPO_4_ (pH 6.0 – 8.0), and 50 mM Na_2_CO_3_-NaHCO_3_ (pH 8.0 - 10.0). The reaction rates were calculated using the absorbance changes at 240 nm within the first 1 min. The concentrations of the enzymes used in the assay were Cat1: 1.06 nM, Cat2: 1.35 nM, Cat3: 3.83 nM, Cat4: 1.11 nM, and Cat5: 10.32 nM.

The catalase activities at different temperatures (30 to 65°C) were measured in 50 mM NaH_2_PO_4_-Na_2_HPO_4_ (pH 7.4). The reaction rates were calculated using the absorbance changes at 240 nm within the first 1 min. The concentrations of the enzymes used in the assay were Cat1: 1.06 nM, Cat2: 1.35 nM, Cat3: 3.83 nM, Cat4: 1.11 nM, and Cat5: 10.32 nM.

The apparent *K*_*m*_ and *k*_*cat*_ values of the five catalases were determined by non-linear least squares fitting using reaction rates with different H_2_O_2_ concentrations (7.5 - 50 mM) at 30°C in 50 mM NaH_2_PO_4_-Na_2_HPO_4_ (pH 7.4). The specific activities of the five catalases were determined using the reaction rates when 16 mM H_2_O_2_ was used as the substrate. The reaction rates were calculated using the absorbance changes at 240 nm within the first 1 min. The concentrations of the holoenzymes (deduced from the detected heme occupancy of each purified catalase) used in the reactions were Cat1: 1.15 nM, Cat2: 1.17 nM, Cat3: 1.26 nM, Cat4: 1.05 nM, and Cat5: 6.05 nM.

### Bioinformatic Analysis of Catalase Homologs

The catalase homologs were searched using standalone BLASTP program (Camacho et al., 2009) with the 21 known catalases reported previously as query sequences (Supplementary Table 3; Zamocky et al., 2008). Pfam search was performed using HMMER 3.3 (Eddy, 2011) with reported Pfam motifs (typical catalase: PF00199, catalase-peroxidase: PF00141, Mn-catalase: PF05067) (Zamocky et al., 2008; El-Gebali et al., 2019). Sequence comparison was performed using EMBOSS 6.5.0 (Rice et al., 2000). Those homologs with Pfam scores lower than the trusted cutoffs of corresponding Pfam motifs were removed, along with those with shorter lengths (typical catalase clade 1: <450 residues, clade 2: <600 residues, clade 3: <450 residues, catalase-peroxidase: <600 residues, Mn-catalase: <180 residues). The phylogenetic trees were constructed by a neighbor-joining method using MEGA X with the default parameters (Kumar et al., 2018). The information on all catalase homologs, including their GenBank accession numbers and sequences, are provided as Supplementary Dataset 1; the phylogenic trees of three families of catalases are provided as Newick files in Supplementary Dataset 2.

## Results

### Phylogenetic Analysis Revealing the Diversity of Catalases in Bacteria

**FIGURE 1:**
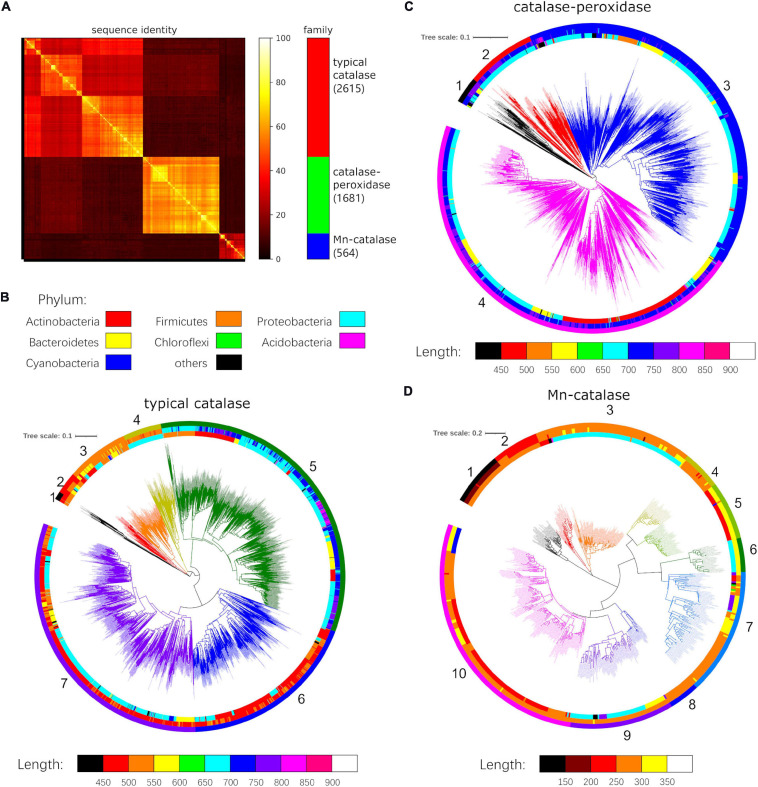
Sequence comparison and phylogenetic analysis of three catalase families. **(A)** Pairwise sequence identities of all 4,860 catalase homologs. Phylogenetic trees of **(B)** typical catalases, **(C)** catalase-peroxidases, and **(D)** Mn-catalases. The inner strip chart indicates the phylum (red: Actinobacteria, orange: Firmicutes, yellow: Bacteroidetes, cyan: Proteobacteria, green: Chloroflexi, blue: Cyanobacteria, magenta: Acidobacteria, black: others). The middle strip chart denotes the protein length with the corresponding color maps. The outer strip chart indicates the groups of each catalase family.

To explore the abundance and diversity of catalases in bacteria, we carried out a virtual survey of 13,360 completed bacterial genomes in the NCBI Genomes database (as of Sep 2020) using the 21 well studied catalases covering all three families (Supplementary Table 3) as query sequences. As the numbers of sequenced genomes varied dramatically among different taxa, no more than 10 genomes from each genus were randomly selected as representatives for further analysis. As a result, a total of 2,634 genomes were selected, in which 4,931 catalase homologs were identified. The homologs were further filtered by removing 71 proteins with Pfam scores lower than the trusted cutoffs or with abnormal protein sequence lengths (Supplementary Figure 1). Pairwise sequence comparisons of the final 4,860 catalase homologs clearly showed the three distinct protein families consisting of the typical catalases, catalase-peroxidases, and Mn-catalases (Figure 1A).

We next subjected the proteins of each catalase family to phylogenetic analysis, which revealed diverse groups within each family (Figure 1). In the phylogenetic tree, 2,615 typical catalase homologs fell into seven groups (group 1-7, Figure 1B). The proteins of group 5 have apparently larger sizes ranging from 600 to 850 residues. They showed relatively lower sequence similarity to the homologs from other groups (Supplementary Figure 2A). The proteins from groups 6 and 7 have 450-550 residues. The group 6 catalase homologs are mainly from Actinobacteria, while a small portion of them are from Proteobacteria and Firmicutes. However, the majority of group 7 proteins are from Proteobacteria, with a small minority of them coming from Bacteroidetes and Firmicutes. The proteins of groups 2, 3, and 4 are predominantly 500-600 residues in size. Among them, the group 4 catalase homologs are from Proteobacteria, while the proteins of groups 2 and 3 come from various phyla (Figure 1B). The proteins from group 1 are shorter (450-500 residues), and display relatively lower sequence identity to the homologs from other groups of this family (Supplementary Figure 2A). The proteins of this group are mainly from Firmicutes.

Phylogenetic analysis of the 1,681 catalase-peroxidase homologs appeared to form four major groups (Figure 1C). However, these groups are closely related, as they are all approximately 700-850 residues in size, and share high sequence identity (Figure 1C and Supplementary Figures 1B, 2B), forming a tight catalase-peroxidase protein family. Among them, the proteins in group 3 are mainly from Proteobacteria, and a minority of them come from Bacteroidetes and Firmicutes. The proteins in group 4 are from Actinobacteria, Proteobacteria, and Bacteroidetes. The proteins in group 1 come from various phyla, while group 2 proteins are mainly from Proteobacteria.

For the Mn-catalase family, the 564 homologs clearly formed ten groups, and their inter-group sequence identities were typically lower than 40% (Figure 1D and Supplementary Figure 2C). The proteins from groups 1 and 2 are significantly shorter in length (group 1: 150-200 residues, group 2: 200-250 residues), and these proteins are from Firmicutes. Most proteins of group 3 and 4 have medium lengths (250-300 residues), and are mainly from Proteobacteria and Firmicutes, respectively. In contrast, the proteins of groups 5 and 6 are larger (300-350 residues) than others, and they are shown to be from Actinobacteria and Proteobacteria, respectively (Figure 1D). The proteins of group 7 differ in length and taxa distribution. The group 8 protein members are mainly from *Carnobacterium* and *Lactobacillus*, while those of group 9 are from Proteobacteria, Bacteroidetes, and *Deinococcus*. The proteins of group 10 vary over a broad range in length, and their host strains include Actinobacteria, Firmicutes, and Cyanobacteria.

### The Taxa Distribution of Catalases

**FIGURE 2:**
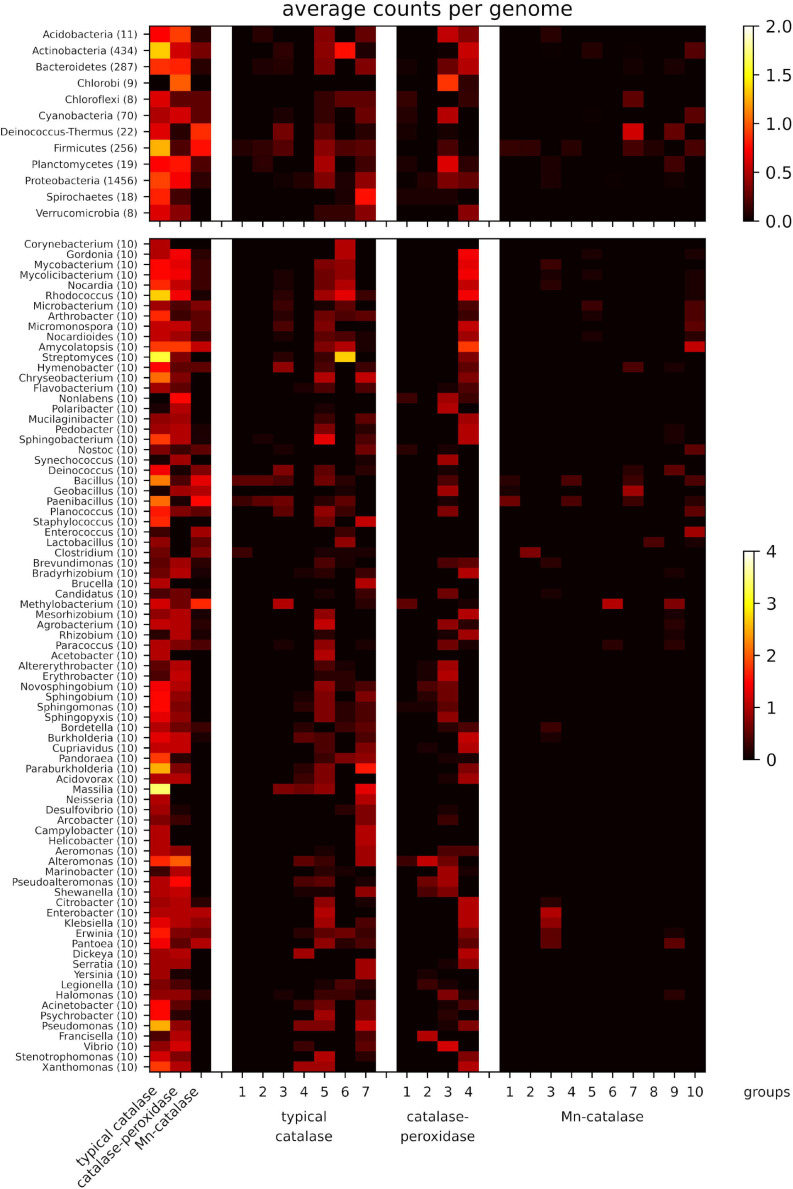
Average counts per genome of the three catalase families (left part) and the different groups (right part) for well-sampled phyla (upper part) and genera (lower part). Data from those phyla with less than 5 genomes and genera with less than 10 genomes are not shown.

We first surveyed and compared the richness and diversity of catalases among different bacteria at the phylum level. The distribution patterns of the three catalase families varied among different phyla (Figure 2). Overall, the typical catalases are widely distributed except in Chlorobi, and the catalase-peroxidases are present in many phyla, while Mn-catalases mainly exist in Deinococcus-Thermus and Firmicutes. The phyla, such as Acidobacteria, Actinobacteria, Bacteroidetes, Planctomycetes, and Proteobacteria, are rich in typical catalases and catalase-peroxidases but lack Mn-catalases. In contrast, the phylum of Deinococcus-Thermus is rich in Mn-catalases and typical catalases, but not catalase-peroxidases. The majority of catalases in Chlorobi are catalase-peroxidases, while the typical catalases are the ones dominant in Spirochetes. Cyanobacteria and Chloroflexi have relatively fewer catalases than other phyla. The shortage of typical catalases in Cyanobacteria has been also observed previously (Zamocky et al., 2008; Johnson and Hug, 2019).

We then further analyzed the distributions of three catalase families in 81 well-sampled genera (each genus with ten selected genomes). A notable high frequency of typical catalases was observed in soil bacteria *Streptomyces*, *Massilia*, *Rhodococcus*, and the pathogenic bacteria *Pseudomonas* (Figure 2). Indeed, all the selected genera under the phylum of Actinobacteria are high in typical catalases (mainly groups 5 and 6), and majority of them are also rich in catalase-peroxidase (mainly group 4) (Figure 2). Some genera of Actinobacteria also contain Mn-catalases, such as *Amycolatopsis*. The *Bacillus* and *Paenibacillus* strains are rich in various groups of typical catalases and Mn-catalases, while their close relatives, the thermophilic *Geobacillus* strains, mainly contain catalase-peroxidases and Mn-catalases. The *Staphylococcus* strains are rich in typical catalases (groups 5 and 7) but lack of catalase-peroxidases and Mn-catalases. The facultative or strict anaerobes under the Firmicutes phylum, including *Enterococcus*, *Lactobacillus*, and *Clostridium*, are also short of catalase-peroxidases but rich in typical catalases and Mn-catalases. The representative opportunistic pathogenic bacteria, such as *Enterobacter*, *Klebsiella*, and *Proteus* strains, possess all three catalase families, including the typical catalases (mainly group 5), catalase-peroxidases (group 4), and Mn-catalases (group 3). However, the richness in all the three catalase families is not observed in other pathogenic bacteria like *Yersinia* and *Pseudomonas* strains.

### A Comprehensive Survey of Catalases in *Streptomyces*

In particular, we were interested to know the abundance and diversity of catalases in *Streptomyces* strains, which have complex life cycles and are the prolific producers of natural products with therapeutic applications (Bentley et al., 2002; Flärdh and Buttner, 2009; Musiol-Kroll et al., 2019). To get an overarching view of the distribution of catalases in *Streptomyces*, we extracted all the catalase homologs in 271 annotated *Streptomyces* genomes available in NCBI Genomes database, resulting in 811 typical catalase homologs and 268 catalase-peroxidase homologs, with no Mn-catalase homologs identified. In other words, there are 2.99 typical catalases and 0.99 catalase-peroxidases on average in each *Streptomyces* genome. Specifically, the catalase homologs in four well-characterized antibiotic-producing *Streptomyces* strains, including *S. coelicolor* A3(2), *S. venezuelae* ATCC 10712 (ISP5230), *S. avermitilis* MA-4680, and *S. rimosus* ATCC 10970, are listed in Supplementary Table 4. It is worth noting that, although an average of 0.99 catalase-peroxidase homolog per genome was found in *Streptomyces*, it was clear that some *Streptomyces* strains contain no catalase-peroxidase (such as *S. avermitilis* MA-4680 and *S. rimosus* ATCC 10970), whereas some other strains can have more than one homolog (like *S. venezuelae* ATCC 10712; Supplementary Table 4).

**FIGURE 3:**
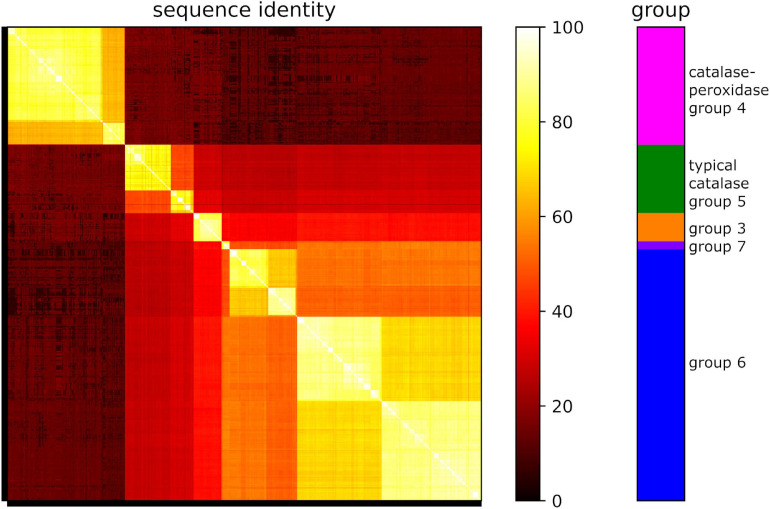
Protein sequence identities of catalase homologs from 271 *Streptomyces* genomes. Groups are shown in different colors. Orange: typical catalase group 3; green: typical catalase group 5; blue: typical catalase group 6; violet: typical catalase group 7; magenta: catalase-peroxidase group 4.

Remarkably, sequence analyses revealed that all the identified 268 catalase-peroxidase homologs belong to group 4 (Figure 3). For the 811 typical catalase homologs, the majority of them belong to group 6 (71%), with the remaining homologs falling into groups 5 (19%), 3 (8%), and 7 (3%). This distribution pattern was consistent with that of the ten randomly selected *Streptomyces* genomes (Figure 2). As shown in Figure 3, the group 5 and 6 typical catalases in *Streptomyces* may be further divided into two subgroups based on sequence analysis.

### Functional Characterization of Five Catalases From *S. rimosus* ATCC 10970

The above bioinformatics analysis revealed the richness of typical catalase homologs in *Streptomyces*. To verify the predicted catalase function of these homologs, we selected and characterized all five typical catalase homologs (Cat1-5) identified in *S. rimosus* ATCC 10970, the oxytetracycline producing strain (Zhang et al., 2006). The five proteins with N-terminal His_6_-tags were overproduced in *E. coli* BL21(DE3), and purified to homogeneity (Supplementary Figure 3). The purified proteins were reddish brown in solution, and their UV-vis spectra showed characteristic Soret bands at 405 nm (Supplementary Figure 4), supporting that they were heme-binding enzymes. All five enzymes could catalyze the decomposition of H_2_O_2_, as the absorbance at 240 nm of the reaction mixtures decreased continuously for all five enzymes while such an absorbance change was not observed for the reaction without enzymes (Supplementary Figure 5). These results confirmed that all five proteins are catalases. The specific activities of these catalases were determined, in which the Cat5 (group 5, Clade 2) showed relatively low activity (Table 1). Overall, the specific activities of these typical catalases were comparable to those of reported catalases (Switala and Loewen, 2002; Sooch et al., 2014).

**TABLE 1:** The catalytic activities and kinetic parameters of the five typical catalases from *S. rimosus* ATCC 10970.

Name	Protein ID	Molecular weight (kDa)	Specific activity * (×10^5^ U/mg holoenzyme)	*K*_*m*_ (mM)	*k*_*cat*_ * (×10^5^ s^–1^)	*k*_*cat*_/*K*_*m*_ * (×10^4^ s^–1^ mM^–1^)
**Cat1**	QEV74945.1	56.69	1.53 ± 0.08	53.5 ± 9.4	6.55 ± 0.72	1.22 ± 0.25
**Cat2**	QEV75671.1	64.95	2.21 ± 0.03	24.8 ± 4.3	6.09 ± 0.51	2.46 ± 0.48
**Cat3**	QEV75878.1	56.63	1.70 ± 0.20	35.1 ± 4.5	5.13 ± 0.36	1.46 ± 0.22
**Cat4**	QEV76009.1	57.46	2.24 ± 0.10	20.1 ± 1.4	4.70 ± 0.15	2.33 ± 0.18
**Cat5**	QEV79658.1	79.43	0.237 ± 0.006	44.7 ± 6.3	1.24 ± 0.11	0.276 ± 0.046

*The specific activities and kinetic parameters were calculated based on the concentrations of holoenzymes (deduced from the detected heme occupancy of each purified catalase).

**FIGURE 4:**
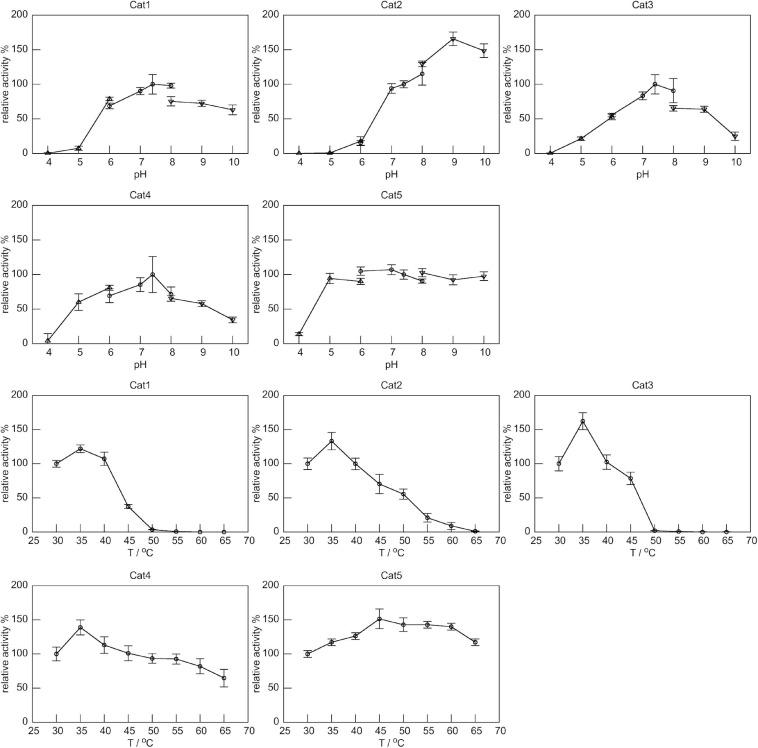
The relative specific activities of five catalases under different pH and temperatures. Specific activity of each catalase at 30°C and pH 7.4 was set as 100%. The catalytic activities at different pH values were determined at 30°C using several buffer systems: 50 mM NaAc-HAc, pH 4.0 – 6.0 (up triangle), 50 mM NaH_2_PO_4_-Na_2_HPO_4_, pH 6.0 – 8.0 (circle), and 50 mM Na_2_CO_3_-NaHCO_3_, pH 8.0 - 10.0 (down triangle). Activities at different temperatures were determined at pH 7.4.

We then further analyzed the properties of these catalases by evaluating the optimal reaction pH and temperature for each of them. The Cat1, Cat3, and Cat4, all belonging to group 6, showed optimal pH at 7.4, and their activities decreased significantly when pH changed (Figure 4). Cat2 (group 3) showed high activities under alkaline pHs, with an optimal pH of 9.0. The Cat1-4 showed highest activities at 35°C, and their activities decreased significantly above 35°C. Surprisingly, Cat5 (group 5) showed nearly constant activity over a broad pH range of 5.0-10.0, and remained active at temperatures from 30 to 65°C (Figure 4). The thermostabilities of five catalases were also characterized by circular dichroism (CD) spectroscopic analysis. All five catalases showed negative peaks at 208 and 222 nm (Supplementary Figure 6), which corresponded to the α-helices of the conserved α + β catalase fold (Nicholls et al., 2000; Chelikani et al., 2004; Díaz et al., 2012). The thermal denaturation processes of five catalases were monitored by the CD signals at 222 nm (Supplementary Figure 6). Among them, Cat1 (Tm: 52°C) and Cat3 (Tm: 53°C) were less resistant to heat, while Cat4 (Tm: 75°C) displayed moderate heat tolerance. Surprisingly, although Cat2 showed complete loss of activity at 65°C, no significant change of the CD signal at 222 nm was observed up to 80°C. These results indicated that, under the above assay conditions, the tertiary structure of Cat2 was likely damaged, but most of the α-helix secondary structures remained unchanged. Cat5 displayed excellent thermostability up to 80°C, which explained why this enzyme remained catalytically active at 65°C as shown in Figure 4.

Due to the two-stage reaction mechanism, catalases did not follow Michaelis-Menten kinetics except at low H_2_O_2_ concentrations (Switala and Loewen, 2002; Zamocky et al., 2008). Therefore, the apparent *K*_*m*_ and *k*_*cat*_ values for the five catalase reactions were determined at low substrate concentrations (not more than 50 mM) (Table 1 and Supplementary Figure 7). Among them, Cat5 displayed lower catalytic efficiency. Overall, the kinetic values were comparable to the reported data of known typical catalases (Switala and Loewen, 2002; Jia et al., 2016, 2017).

## Discussion

Catalases are widely distributed in most aerobic organisms and also in some anaerobic microorganisms (von Ossowski et al., 1993; Whittaker, 2012; Zámocký et al., 2012). An early phylogenetic analysis of 20 typical catalases suggested that small subunit typical catalases of animal and fungi were derived from one ancestor, while those catalases of plant originated from another ancestor (von Ossowski et al., 1993). The study also revealed that the typical catalases from bacteria did not cluster together (von Ossowski et al., 1993). The following studies with more catalase proteins showed that bacterial typical catalases fell into three clades (Klotz et al., 1997; Klotz and Loewen, 2003), which was further supported by later molecular evolution analyses using more catalase homologs (Zamocky et al., 2008; Zámocký et al., 2012). The Clade 1 (small subunit) typical catalases were mainly from plants and bacteria (Firmicutes and Proteobacteria), the Clade 3 (small subunit) typical catalases were from animal, fungi, archaea, and various bacteria, while the Clade 2 (large subunit) typical catalases were shown to be from bacteria and fungi (Zámocký et al., 2012). Molecular evolution analysis showed that the catalase-peroxidases fell into two clades, the (main) Clade 1 and the (minor) Clade 2, while the Mn-catalases were separated into five clades (Zámocký et al., 2012).

In bacteria, catalases are the key players in the defense against oxidative stress (Zámocký et al., 2012; Johnson and Hug, 2019; Kim et al., 2019). In addition, they also participate in many other cellular processes, such as cell development and differentiation, and production of metabolites (Cho et al., 2000; Aguirre et al., 2005; Matsuura et al., 2014; Montibus et al., 2015; Bibián et al., 2020). In this study, by mining the abundant genomic information available in the GenBank database, we were able to know the overall abundance, diversity, and distribution of catalases in bacteria. Compared to previous studies (von Ossowski et al., 1993; Klotz et al., 1997; Klotz and Loewen, 2003; Chelikani et al., 2004; Zamocky et al., 2008; Zámocký et al., 2012), our study focused on bacteria and included thousands of catalase homologs for analysis. Unlike the catalase-peroxidase family in which the proteins were closely related, both typical catalase and Mn-catalase families could be classified into distinct groups.

Based on sequence similarities, the typical catalase groups 3 and 4, group 5, and groups 6 and 7 in our study, might correspond to the previous Clade 1 (small subunit), Clade 2 (large subunit), and Clade 3 (small subunit), respectively. The catalase-peroxidase groups 3 and 4 corresponded to the previously identified Clade 1 (main clade), and the group 2 seemed to be correlated to the Clade 2 (minor clade). The Mn-catalase groups 4 and 5, group 7, and groups 8-10, appeared to correspond to the previously reported Clade 2, Clade 1, and Clade 3 of Mn-catalases, respectively, while group 3 seemed to correspond to both Clades 4 and 5 (Zámocký et al., 2012). Meanwhile, the homologs in typical catalase group 1 and 2, and Mn-catalase group 1 and 6 showed relatively low sequence similarities to known catalases in RedoxiBase, a database containing the catalases used for analyses in previous studies (Zámocký et al., 2012; Savelli et al., 2019; Supplementary Figure 8).

In particular, we paid great attention to the *Streptomyces* strains, which are well-known for their complicated cell cycles and capability for producing various valuable secondary metabolites (Bentley et al., 2002; Flärdh and Buttner, 2009; Musiol-Kroll et al., 2019). An extreme richness of typical catalases and catalase-peroxidases was observed in *Streptomyces*. On average, each *Streptomyces* strain contains 2.99 typical catalases and 0.99 catalase-peroxidases. But remarkably, there were no Mn-catalases found in any of the *Streptomyces* genomes. Sequence comparison of these catalase homologs clearly showed several subgroups of typical catalases (Figure 3), which might be the result of gene duplication. To support our bioinformatic analyses, the five typical catalase homologs identified in *S. rimosus* ATCC 10970 were characterized *in vitro*. All of them showed the catalytic activities of H_2_O_2_ decomposition. Among them, Cat5 (group 5, Clade 2) had the lowest specific activity but showed remarkable pH and thermal tolerances. The typical catalases (Clade 2) from *Aspergillus niger* and *E. coli* were also reported to show low activities, and in addition, the catalase from *E. coli* exhibited good thermal stability (Switala and Loewen, 2002). Another typical catalase (Clade 2) was isolated from *Aspergillus terreus* MTCC 6324, and it was extremely active and stable in broad pH range (4-12) and at temperatures up to 90°C (Vatsyayan and Goswami, 2016).

Taken together, our results set the stage for further studies of bacterial catalases to understand their roles in the adaptation of host strains to internal and external environmental changes. Furthermore, previous studies showed that typical catalases were involved in the regulation of development and differentiation of streptomycetes, and affected the production of several secondary metabolites (Cho et al., 2000; Beites et al., 2011), suggesting the manipulation of these catalases as a potential approach to modulate the secondary metabolite production in strains like streptomycetes.

## Data Availability Statement

The datasets presented in this study can be found in online repositories. The names of the repository/repositories and accession number(s) can be found in the article/Supplementary Material.

## Author Contributions

GP, KF, and ZL conceived the idea. FY, YX, LX, and SY performed the experiments. FY, KF, and GP analyzed the data and wrote the manuscript with the input of all authors. KF, FY, and HW carried out the bioinformatics analyses. All authors edited the manuscript and approved its final version.

## Conflict of Interest

The authors declare that the research was conducted in the absence of any commercial or financial relationships that could be construed as a potential conflict of interest.
