# Supplementary material for: The Richness and Diversity of Catalases in Bacteria
Source: Front Microbiol. 2021 Mar 19;12:645477. doi: 10.3389/fmicb.2021.645477 (PMC8017148; doi:10.3389/fmicb.2021.645477)
Supplement: Supplementary file 1 [file Image_1.PDF]

Supplementary Material

The richness and diversity of catalases in bacteria

Fang Yuan[1,2], Shouliang Yin[1,3], Yang Xu[1], Lijun Xiang[1], Haiyan Wang[1], Zilong Li[1], Keqiang Fan[1*], Guohui Pan[1,2*]

[1]State Key Laboratory of Microbial Resources, Institute of Microbiology, Chinese Academy of Sciences, Beijing, China

[2]University of Chinese Academy of Sciences, Beijing, China

[3]School of Life Sciences, North China University of Science and Technology, Tangshan, Hebei, China

*** Correspondence:**
Keqiang Fan
fankq@im.ac.cn
Guohui Pan
panguohui@im.ac.cn

1 Supplementary Figures and Tables

1.1 Supplementary Figures

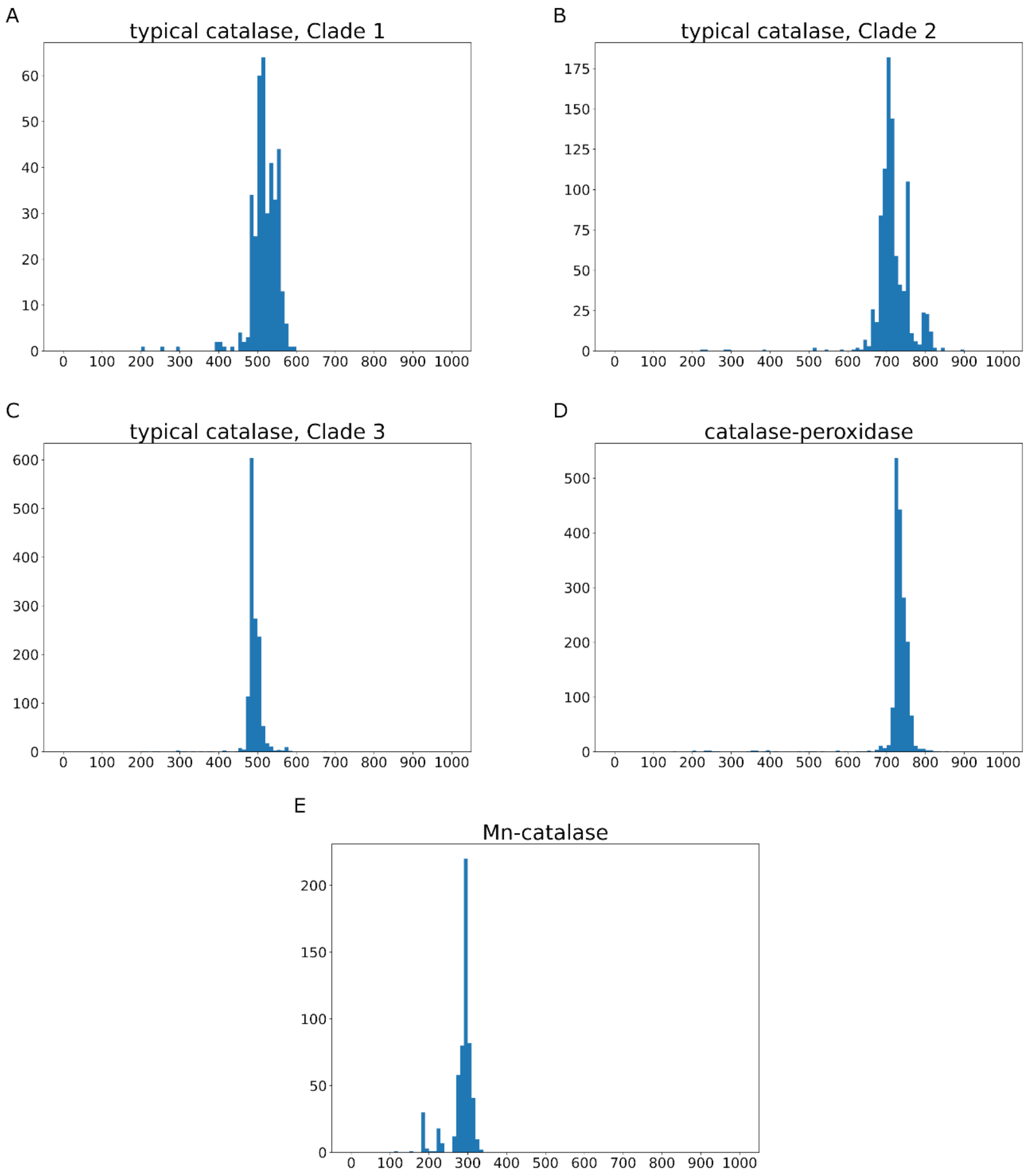

Supplementary Figure 1. Protein length distribution of different catalase families.

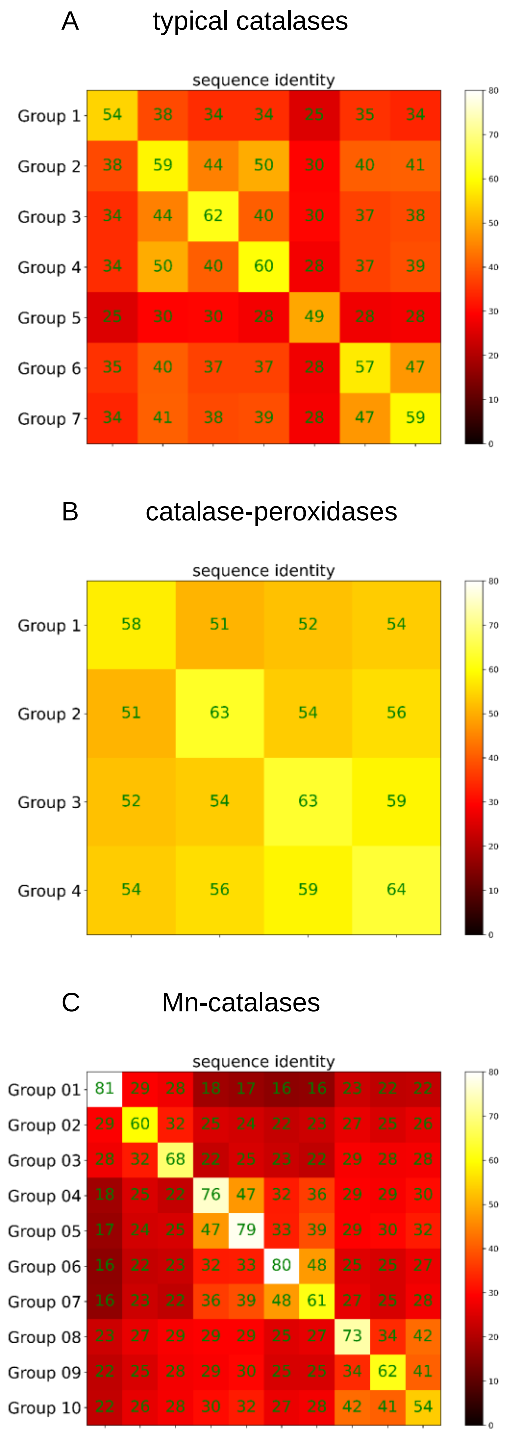

Supplementary Figure 2. The average intra- and inter-group sequence identities for three catalase families.

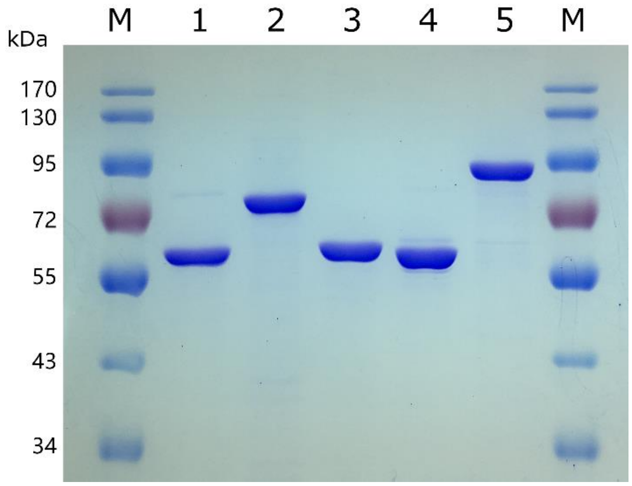

Supplementary Figure 3. The SDS-PAGE of five purified typical catalases (Cat1−5) from *S. rimosus* ATCC 10970. Line 1: His_6-Cat1, 56.69 kDa, Line 2: His_6-Cat2, 64.95 kDa, Line 3: His_6-Cat3, 56.63 kDa, Line 4: His_6-Cat4, 57.46 kDa, Line 5: His_6-Cat5, 79.43 kDa, M: protein marker.

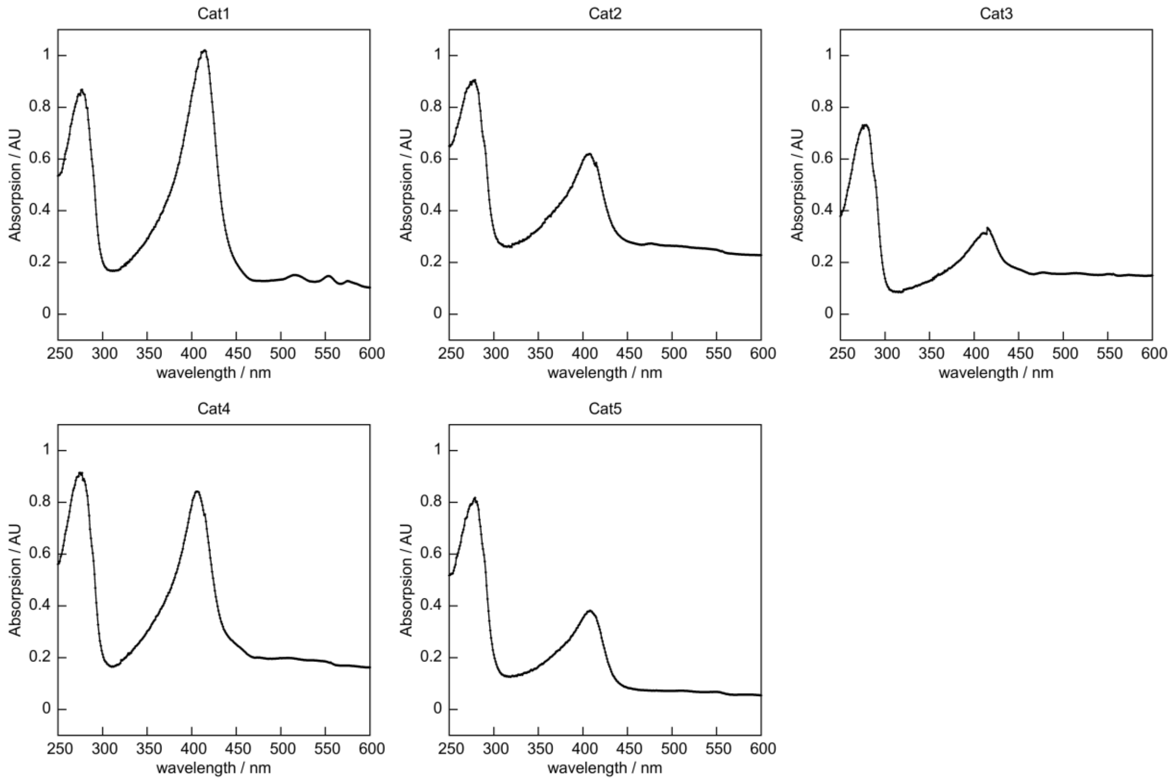

Supplementary Figure 4. UV-vis spectra of five purified catalases. The concentrations of five catalases used for analyses were: Cat1: 8.47 μM, Cat2: 6.93 μM, Cat3: 8.83 μM, Cat4: 8.70 μM, and Cat5: 6.30 μM. The heme concentrations were calculated using absorption at 406 nm (ε_{406} = 102 mM^{-1} cm^{-1}). The heme occupancy of each catalase was the ratio of heme concentration to the corresponding protein concentration. The heme occupancies were determined as Cat1: 1.091, Cat2: 0.867, Cat3: 0.329, Cat4: 0.941, and Cat5: 0.586.

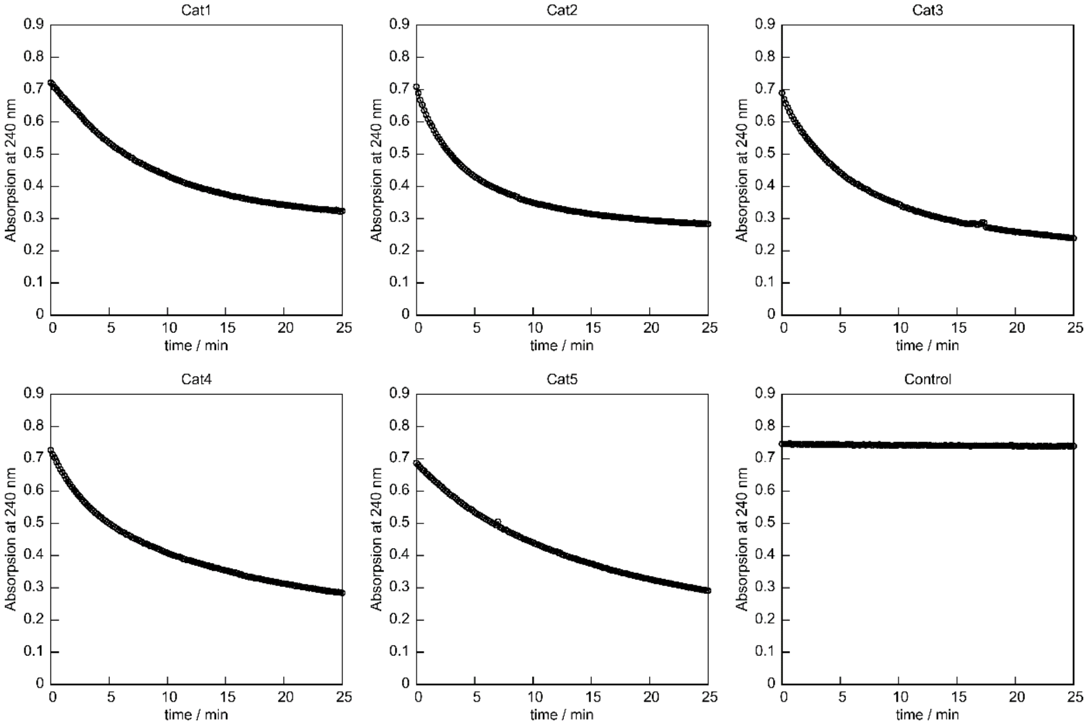

Supplementary Figure 5. The reactions of five catalases monitored by absorbance at 240 nm. The concentrations of five catalases used in the assay were Cat1: 1.06 nM, Cat2: 1.35 nM, Cat3: 3.83 nM, Cat4: 1.11 nM, and Cat5: 10.32 nM.

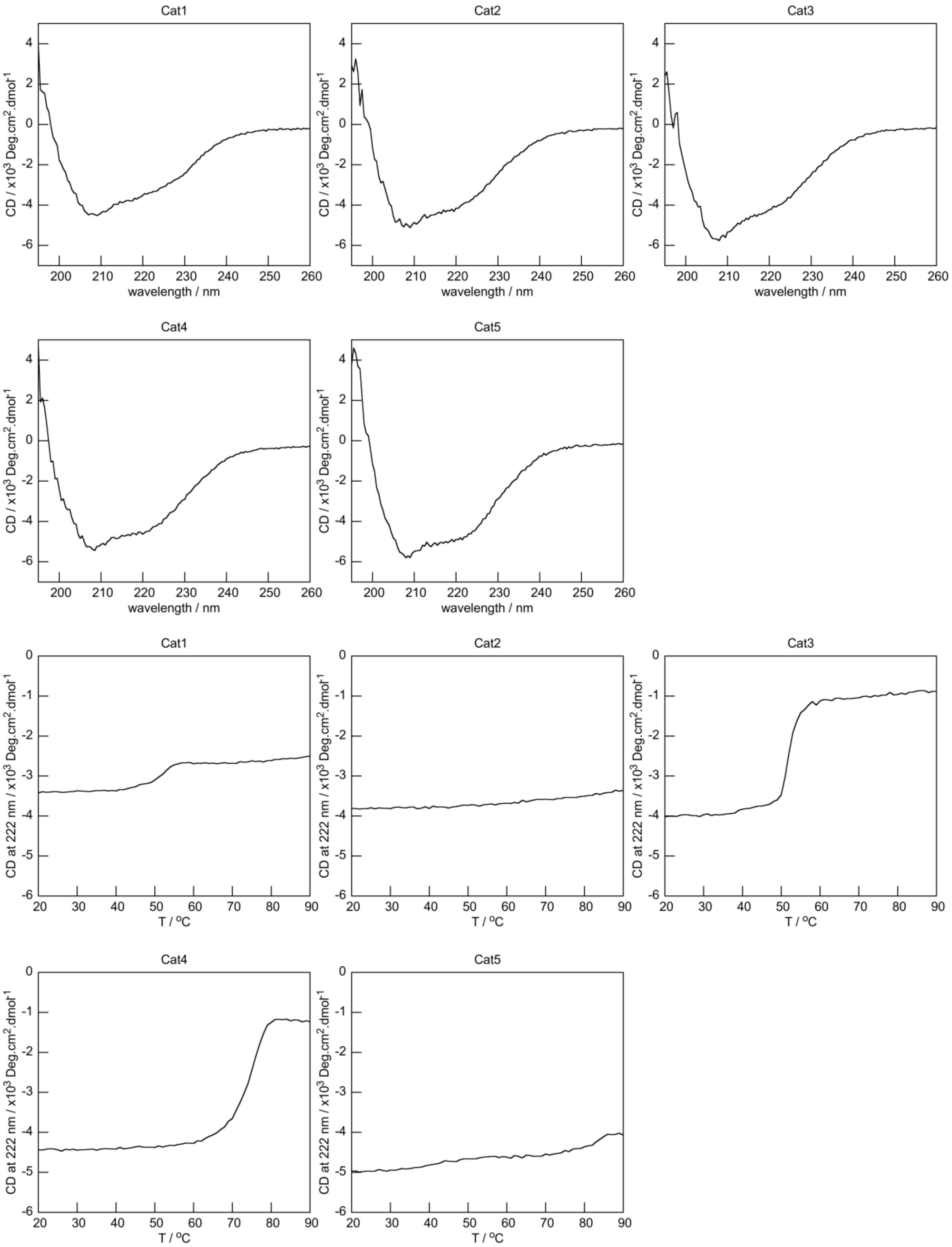

Supplementary Figure 6. CD spectra (at 30 °C) and thermal denaturation curves of five catalases. The concentrations of five catalases used in the assay were: Cat1: 3.67 μM, Cat2: 3.43 μM, Cat3: 3.67 μM, Cat4: 3.13 μM, Cat5: 2.71 μM.

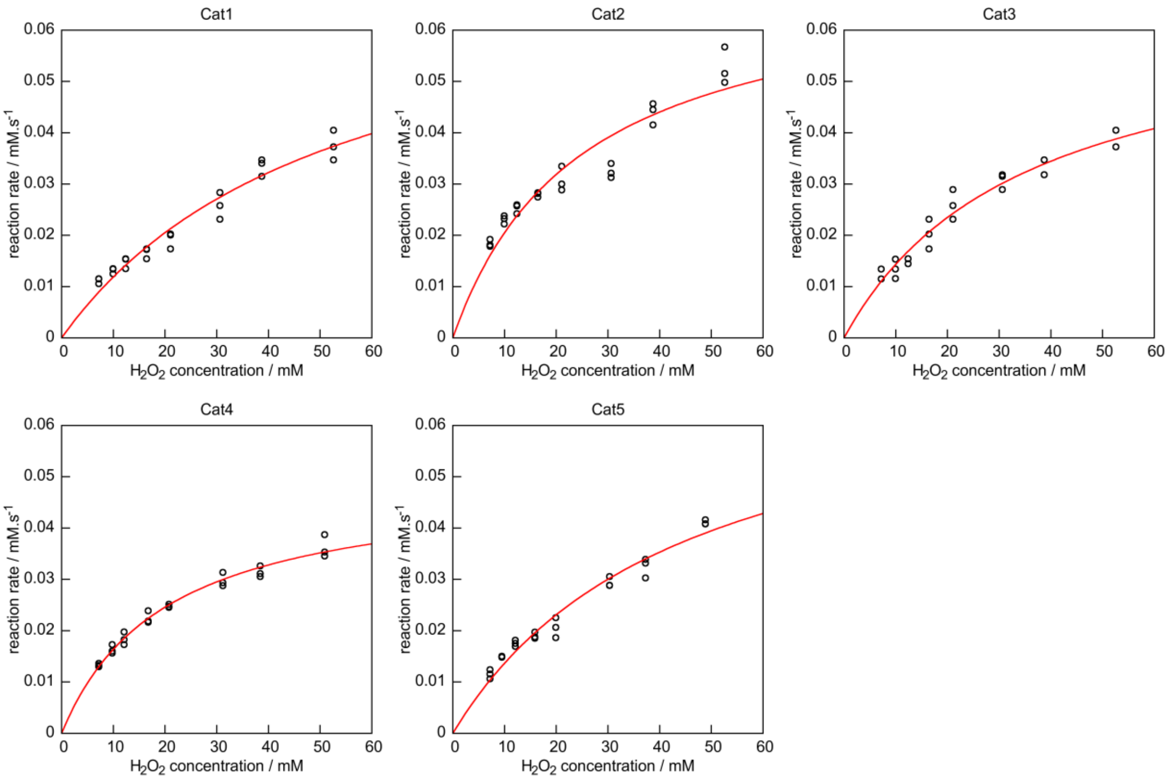

Supplementary Figure 7. Dependence of enzyme velocity on H_2O_2 concentration. In all panels the open circle represents the observed data and the red line represents the theoretical Michaelis–Menten curve determined by nonlinear least squares fitting. The concentrations of the holo-enzymes (deduced from the detected heme occupancy of each purified catalase) used in the reactions were Cat1: 1.15 nM, Cat2: 1.17 nM, Cat3: 1.26 nM, Cat4: 1.05 nM, and Cat5: 6.05 nM.

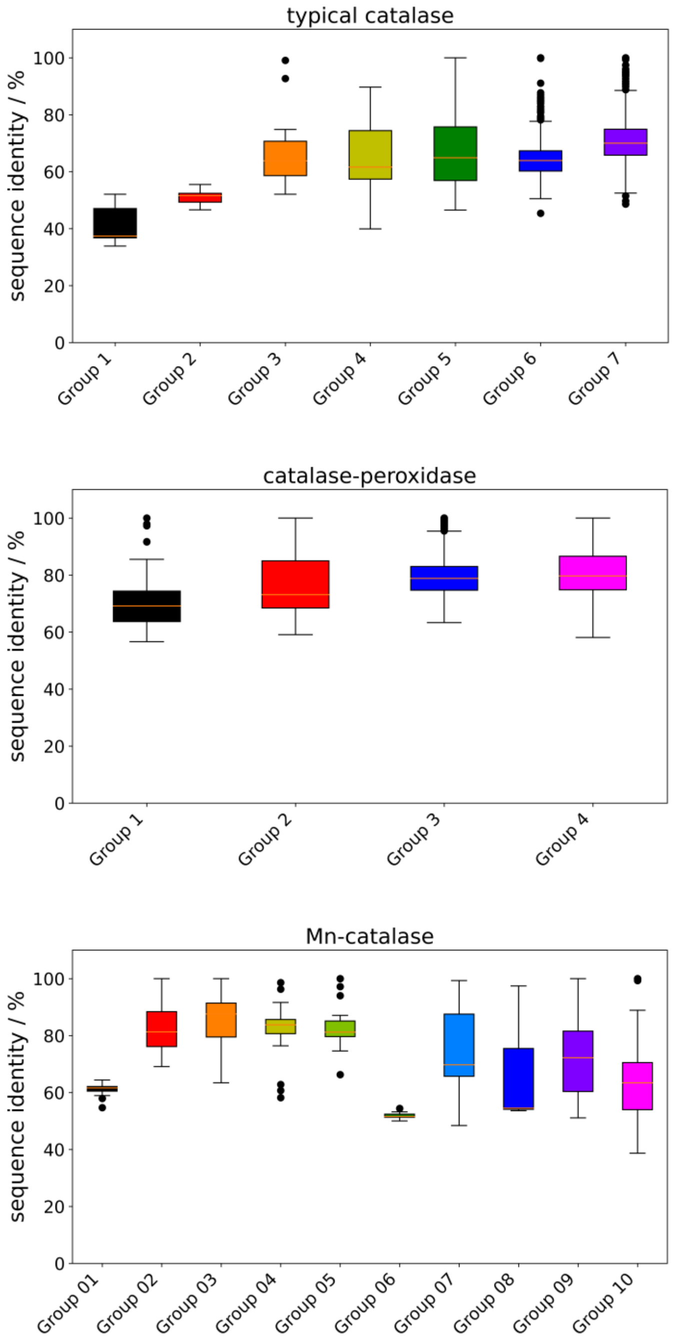

Supplementary Figure 8. The sequence comparisons between the catalase homologs in this study and the catalases in RedoxiBase database (http://peroxibase.toulouse.inra.fr/). Each catalase homolog in this study was compared to all the catalases in RedoxiBase database, and only the highest sequence identity was used for the analysis.

1.2 Supplementary Tables

Supplementary Table 1. Strains and Plasmids used in this study.

Name	Description	Sources
**Strains**		
E. coli JM109	General cloning host for plasmid manipulation	Novagen
E. coli BL21(DE3)	Strain used for the expression of protein	Novagen
**plasmids**		
pET-28a	*E. coli* expression vector; Kana^r	Novagen
pET-28a-cat1	pET-28a containing the coding region of Cat1	This study
pET-28a-cat2	pET-28a containing the coding region of Cat2	This study
pET-28a-cat3	pET-28a containing the coding region of Cat3	This study
pET-28a-cat4	pET-28a containing the coding region of Cat4	This study
pET-28a-cat5	pET-28a containing the coding region of Cat5	This study

Supplementary Table 2. Primers used in this study.

Name	Sequence (5' to 3')	Description
28a-catalase-F1	cggatctcagtggtggtggtggtggtgctcgagTCAGTCCTCGCGCAGCTCGTG	Construction of pET-28a-cat1
28a-catalase-R1	cagcagcggcctggtgccgcgcggcagccatatgCCGAAGCCCACGACGACGCG	
28a-catalase-F2	cggatctcagtggtggtggtggtggtgctcgagTCAGCCCGCGACGGCGTGCCG	Construction of pET-28a-cat2
28a-catalase-R2	gcagcggcctggtgccgcgcggcagccatatgGTGACAGACACAGCGAGTCAGG	
28a-catalase-F3	cggatctcagtggtggtggtggtggtgctcgagTCAGCCGCGCAGGGCCTGGAC	Construction of pET-28a-cat3
28a-catalase-R3	cagcagcggcctggtgccgcgcggcagccatatgGTGTCGGTACAGAGCAGCAC	
28a-catalase-F4	ccggatctcagtggtggtggtggtggtgctcgagTCAGCTGCCGTTGAAGCGCG	Construction of pET-28a-cat4
28a-catalase-R4	agcagcggcctggtgccgcgcggcagccatatgACCAGCTCCGCGCACGACGTC	
28a-catalase-F5	ccggatctcagtggtggtggtggtggtgctcgagTCAGCTGGGCAGCGCCGGAC	Construction of pET-28a-cat5
28a-catalase-R5	agcagcggcctggtgccgcgcggcagccatatgGCCGACCCGAAGCAAGAACAG	

The overlaps sequences used for Gibson assembly are in lower-case.

Supplementary Table 3. The 21 known catalases used as query proteins.

PDB code	Source	Family	Type
1GGE	*Escherichia coli*	Typical catalase	Large subunit, clade 2
1SI8	*Enterococcus faecalis*	Typical catalase	Small subunit, clade 3
2A9E	*Helicobacter pylori*	Typical catalase	Small subunit, clade 3
1M85	*Proteus mirabilis*	Typical catalase	Small subunit, clade 3
1GWE	*Micrococcus lysodeikticus*	Typical catalase	Small subunit, clade 3
1M7S	*Pseudomonas syringae*	Typical catalase	Small subunit, clade 1
2ISA	*Vibrio salmonicida*	Typical catalase	Small subunit, clade 1
2J2M	*Exiguobacterium oxidotolerans*	Typical catalase	Small subunit, clade 1
1A4E	*Saccharomyces cerevisiae*	Typical catalase	Small subunit, clade 3
2IUF	*Penicillium vitale*	Typical catalase	Large subunit, clade 2
1SY7	*Neurospora crassa*	Typical catalase	Large subunit, clade 2
1U5U	*Plexaura homomalla*	Typical catalase	Small subunit, fused
8CAT	*Bos taurus* (liver)	Typical catalase	Small subunit, clade 3
1DGB	*Homo sapiens* (erythrocyte)	Typical catalase	Small subunit, clade 3
1ITK	*Haloarcula marismortui*	Catalase-peroxidases	Archae KatG
1UB2	*Synechococcus* PCC 7942	Catalase-peroxidases	Eubacterial KatG
1MWV	*Burkholderia pseudomallei*	Catalase-peroxidases	Eubacterial KatG
1U2K	*Escherichia coli*	Catalase-peroxidases	Eubacterial KatG
1SJ2	*Mycobacterium tuberculosis*	Catalase-peroxidases	Eubacterial KatG
1JKU	*Lactobacillus plantarum*	Mn-catalases	Clade 3
2CWL	*Thermus thermophilus*	Mn-catalases	Clade 1

Supplementary Table 4. Catalase homologs identified in four model strains of *Streptomyces*.

strain	Locus tag	Family	Clade	Group
S. coelicolor A3(2)	SCO0379	Typical catalase	Clade 3	Group 7
	SCO0560	Catalase-peroxidase	Clade 1	Group 4
	SCO0666	Typical catalase	Clade 2	Group 5
	SCO6204	Typical catalase	Clade 3	Group 6
	SCO7590	Typical catalase	Clade 3	Group 6
S. venezuelae ATCC 10712	SVEN_0140	Typical catalase	Clade 3	Group 6
	SVEN_0529	Catalase-peroxidase	Clade 1	Group 4
	SVEN_4860	Typical catalase	Clade 3	Group 6
	SVEN_6086	Typical catalase	Clade 3	Group 6
	SVEN_7254	Typical catalase	Clade 2	Group 5
	SVEN_7337	Catalase-peroxidase	Clade 1	Group 4
S. avermitilis MA-4680	SAVERM_348	Typical catalase	Clade 2	Group 5
	SAVERM_2026	Typical catalase	Clade 3	Group 6
	SAVERM_3052	Typical catalase	Clade 3	Group 6
	SAVERM_3224	Typical catalase	Clade 3	Group 6
S. rimosus ATCC 10970	CP984_07810	Typical catalase	Clade 3	Group 6
	CP984_12150	Typical catalase	Clade 1	Group 3
	CP984_13375	Typical catalase	Clade 3	Group 6
	CP984_14125	Typical catalase	Clade 3	Group 6
	CP984_36155	Typical catalase	Clade 2	Group 5
